# Atlas of Clinical Medicine

**Published:** 1891-09

**Authors:** 


*Atlas of Clinical Medicine.* By BYROM BRAMWELL, M.D., F.R.C.P.E., F.R.S.E. Volume I. Part I. Edinburgh: T. and A. CONSTABLE. 1891.

This is the first instalment of an Atlas to be issued in four parts yearly, each part to consist of 32 folio pages. The whole work will include at least three yearly volumes. The illustrations are to be made a feature of the publication. Each volume is to contain 30 plates, consisting of coloured plates, photogravures, and illustrations in black and white, copied from water-colour drawings and photographs which have been collected by the author.

The letterpress will comprise—(1) Detailed descriptions of many of the diseases illustrated by the plates; (2) Whenever possible, a complete clinical history of individual cases figured in the Atlas; and (3) Articles on medicine, clinical and systematic, with woodcut illustrations.

The present issue deals with the subject of myxœdema, of which a full account is given from the date of its first description; the illustrations consist of three life-like coloured plates, by the aid of which the characteristic *facies* of the disease would at once be recognised by one who was seeing a case for the first time. There is an account of Sporadic Cretinism, admirably illustrated by photographs of typical cases; and the rest of the number is devoted to an exhaustive description of Friedreich's Ataxia, with a number of drawings of the morbid changes in the spinal cord in this disease; photographs of patients, and of the deformity of the foot. Two additional plates are given, one a crayon drawing of Melancholia with fear, and the other a photogravure of a case of Lymphadenoma: these belong to later parts of the work, and are presumably added to give a general idea of the character of the plates. The least that can be said of the illustrations is that they are beautifully executed, and are most artistic and faithful reproductions of the diseases they represent. Dr. Bramwell's name is sufficient guarantee that the clinical descriptions of the cases and systematic accounts of the diseases selected will be well written; and the subjects treated of in this part are fully considered and brought thoroughly up to date. The whole work promises to be most valuable, and one which all who are interested in the progress of medicine will desire to possess. The author has our most cordial wishes for the success of his new undertaking.

